# Les tuméfactions sous mandibulaires: à propos de 42 cas et revue de la literature

**DOI:** 10.11604/pamj.2014.18.302.4033

**Published:** 2014-08-14

**Authors:** Youssef Darouassi, Mohamed Mliha Touati, Mehdi Chihani, Karim Nadour, Haddou Ammar, Brahim Bouaity

**Affiliations:** 1 Service d'Oto-Rhino-Laryngologie et Chirurgie Cervico-faciale, Hôpital Militaire Avicenne, Marrakech, Maroc

**Keywords:** tuméfactions sous mandibulaires, type histologique, traitement, Submandibular swellings, histological type, treatment

## Abstract

Les tuméfactions de la région sous mandibulaire sont une entité pathologique fréquente, caractérisées par un tableau clinique et une démarche diagnostique stéréotypée, et une unicité presque constante au plan chirurgical. Notre travail est une étude rétrospective sur une période de 5 an d'une série consécutive de 42 cas de tuméfactions sous mandibulaires qui ont été colligés au service d'oto-rhino-laryngologie et de chirurgie cervico-faciale de l'hôpital militaire Avicenne de Marrakech. L’âge moyen des patients était de 41 ans. Une prédominance masculine a été retrouvée. Le tableau clinique a été dominé par la tuméfaction sous mandibulaire. Tous les patients ont bénéficié d'une échographie cervicale. La tomodensitométrie cervicale a été réalisée chez 7 patients. La première étiologie était la sous maxillite chronique lithiasique dans 39,02% des cas. Le traitement était chirurgical dans tous les cas. En comparant les résultats avec ceux rapportés dans la littérature, nous allons discuter les différents aspects diagnostiques et thérapeutiques des étiologies les plus fréquentes. Les tuméfactions de la région sous mandibulaire sont fréquentes, nécessitant une démarche diagnostique rigoureuse et une prise en charge thérapeutique adaptée.

## Introduction

Les tuméfactions de la région sous mandibulaire sont une entité pathologique relativement rare, caractérisées par un tableau clinique très stéréotypé, un chapitre diagnostic peu riche et une unicité presque constante au plan chirurgical. Elles font de la glande sous maxillaire un domaine dont l’étude à part mérite quelques intérêts. Le but de notre travail est d’étudier à travers une série de patients et une revue de la littérature l'aspect clinique, thérapeutique et évolutif des tuméfactions sous mandibulaires.

## Méthodes

Notre étude est rétrospective, portant sur 42 patients ayant présenté une tuméfaction sous mandibulaire, qui ont été colligés au service d'oto-rhino-laryngologie et de chirurgie cervico-faciale de l'hôpital militaire Avicenne de Marrakech, durant une période de cinq ans, allant du 1^er^ Janvier 2006 au 31 Décembre 2010. Pour cela, une fiche d'exploitation a été établie, visant à préciser les données cliniques, paracliniques, histologiques, thérapeutiques et évolutives.

## Résultats

L’âge moyen des patients était de 41 ans avec des extrêmes allant de 16 à 75 ans. Une prédominance masculine a été retrouvée (35 hommes pour 7 femmes). Le tabagisme était l'antécédent le plus fréquent, retrouvé dans 35,71% des cas, suivi par l'alcoolisme dans 14,28%. Le tableau clinique a été dominé par la tuméfaction sous mandibulaire, retrouvée chez tous les patients, suivie par la douleur dans 28,57%. La durée d’évolution des signes cliniques variait de 1 mois à 16 ans avec une moyenne de 2,6 ans. La localisation de la tuméfaction était prédominante à gauche dans 59,52% des cas, avec un diamètre inferieur à 4 cm chez la majorité des patients soit 92,85%. Dans notre série on avait noté des sécrétions pathologiques par le canal de Wharton chez 3 cas. Tous les patients avaient bénéficié d'une échographie cervicale.

L'orthopantomogramme et le défilé maxillaire ont été réalisés chez six patients, tandis que la tomodensitométrie cervicale a été réalisée chez 7 patients. L’échographie avait révélé une lithiase salivaire dans 50% des cas, suivie par les adénopathies dans 19,04%, des images évoquant une tumeur dans 19,04%, un abcès dans 4,76%, tandis qu'elle était normale dans 7,14% des cas. L'orthopantomogramme et le défilé maxillaire avaient objectivé une opacité de tonalité calcique au niveau de la région sous mandibulaire dans 50% des cas. La tomodensitométrie cervicale avait objectivé une lithiase sous maxillaire chez 42,85% des patients, un processus tumoral dans 42,85%, et une image de sous maxillite dans 14,28%.

Le traitement était chirurgical chez tous les patients, avec sous maxillectomie dans 90,24% des cas, cervicotomie dans 9,75% et traitement conservateur dans un cas (2,38%). Le curage ganglionnaire a été envisagé chez 2 patients (4,76%). L'examen extemporané a été réalisé chez 4 patients (9,25%). Un seul patient avait présenté en postopératoire une collection cervicale qui a été drainée. L'examen histologique a été dominé par la sous maxillite chronique lithiasique dans 39,02% des cas. Deux patients avaient bénéficié d'un traitement complémentaire: la radiothérapie pour un patient qui a présenté une métastase de carcinome épidermoïde de la loge amygdalienne; et radiothérapie associée à la chimiothérapie pour un patient qui avait présenté un lymphome T. Les patients dont l'histologie avait confirmé la tuberculose, ont été adressés au centre de diagnostic spécialisé de la tuberculose pour débuter le traitement antibacillaire. L’évolution a été marquée par une amélioration de la symptomatologie clinique, rétablissement de l’état général et reprise du poids.

## Discussion

La lithiase est la pathologie la plus fréquente de la région sous mandibulaire, pour Coumel [1], elle représentait 56%, contre 40,4% dans notre série. Dans la littérature, la lithiase s'observe à tout âge; alors que dans notre étude, elle variait surtout entre 40 et 70 ans. Pour Goudal [2], la tuméfaction sous mandibulaire était le symptôme le plus fréquent, suivie par les manifestations infectieuses. Dans notre série, la tuméfaction sous mandibulaire avait été notée dans 100% des cas, suivie par les manifestations mécaniques dans 94%. L’échographie est actuellement le premier examen à envisager devant la pathologie salivaire, qui doit être pratiquée de façon systématique et méthodique [2].

Tous les patients de notre série avaient bénéficié d'une échographie, celle-ci avait évoqué la lithiase dans 100% des cas. Pour Goudal [2], sur 210 cas de lithiases sous mandibulaires, la sous maxillectomie était pratiquée chez 83 patients, alors que dans notre étude la majorité des patients avaient bénéficié d'une sous maxillectomie (94,11%). L'ablation de calcul par voie buccale, peut être réalisée sous anesthésie locale ou générale, selon le siège du calcul. Cette thérapeutique a été réalisée chez un seul patient de notre série. Pour Goudal [2], il avait noté sur 83 sous maxillectomies, des complications dans 31 cas (37,3%). Pour Coumel [1], sur 50 sous maxillectomies, les complications étaient notées dans 31 cas, dominées par les infections postopératoires; cependant aucune complication postopératoire n'a été notée dans notre série.

La localisation sous mandibulaire de la tuberculose est fréquente et représente 37% des adénopathies (ADP) cervicales tuberculeuses [3]. Dans notre série, les ADP tuberculeuses représentaient 33% de la pathologie de la région sous mandibulaire, occupant ainsi la deuxième étiologie des tuméfactions sous mandibulaires après la lithiase. Pour Marland [4] 2/3 des sujets avaient un âge de 20 à 30 ans, alors que dans notre série la tranche d’âge entre 20 et 40 ans avait prédominé avec 64,2%. Pour Schaad [5], au cours des ADP à mycobactéries atypiques, des signes généraux mineurs ont été observés dans 7,2% des cas. La fistulisation cutanée d'une ADP est considérée comme très en faveur d'une étiologie tuberculeuse, cependant dans les ADP cervicales tuberculeuses, la fistulisation était notée pour Marland [4] dans 11% des cas, et pour Schaad [5] dans 13,4%. Aucun patient de notre étude n'avait présenté de signes généraux (amaigrissement, anorexie, fièvre, sueurs nocturnes) ou de fistulisation cutanée. La mise en évidence du bacille de koch (BK) dans les lésions ganglionnaires peut se faire de 2 manières: soit par la recherche du BK dans le liquide de ponction ganglionnaire ou dans les ganglions prélevés [3]. La chirurgie est discutable, cependant seule l'exérèse de l'ADP donne une confirmation bactériologique et/ou anatomopathologique dans 100% des cas [3]. Le traitement comporte l'exérèse des ganglions atteints associée aux antibacillaires [3].

Dans notre étude, les gestes chirurgicaux étaient à visée diagnostique, tous les patients avaient bénéficié d'une sous maxillectomie. Les patients, après résultat histologique, étaient adressés au centre de diagnostic spécialisé de la tuberculose pour débuter le traitement antibacillaire. La localisation de la tuberculose au niveau des glandes salivaires est rare, une centaine de cas seulement a été rapportée dans la littérature, dont moins de 40 cas concernent la glande sous-mandibulaire [6]. La série la plus documentée est celle de Redon [6] datant de 1955 portant sur 24 cas. La tuberculose de la glande sous mandibulaire avait été diagnostiquée chez deux patients de notre série (4,76%). Les tumeurs des glandes sous mandibulaires sont rares et fréquemment groupées avec les autres tumeurs des glandes salivaires [7]. Les données concernant leurs fréquences sont variables selon les auteurs (Tableau 1) [7–9]. En 2009, elles représentaient entre 2% et 6,5% des tumeurs de la tête et du cou, 63% de ces tumeurs concernaient les glandes salivaires principales [7]. L'imagerie occupe une place importante dans le diagnostique des tumeurs des glandes salivaires, dominée par l'imagerie par résonance magnétique (IRM), alors que les radiographies sans préparation, la sialographie et la scintigraphie ne sont plus utilisées [10]. L’échographie avait été longtemps considérée comme un examen clé de l'exploration des tumeurs des glandes salivaires, ce qui n'est plus le cas [10]. L'IRM est l'examen le plus performant pour l'exploration des tumeurs des glandes salivaires, une récente revue a été publiée par Halimi et al [10].

**Tableau 1:** Fréquence des tumeurs de la glande sous mandibulaire

Auteurs	Année	Nombre de cas	Bénignes %	Malignes %
Manipoud [8]	1995	10	70	30
Chua [9]	2010	101	79	21
Becerril-Ramírez [7]	2011	22	86	14
Notre série	2013	7	71	29

Au niveau histologique, les tumeurs retrouvées sont épithéliales dans environ 95% des cas, bénignes pour 66% d'entre elles. Les tumeurs bénignes épithéliales les plus fréquemment retrouvées sont les adénomes pléomorphes pour 50% des cas [10]. Dans notre travail, l'adénome pléomorphe occupe la première place dans la pathologie tumorale, soit 43% des tumeurs des glandes sous mandibulaires. Il survient préférentiellement chez le sexe féminin (ratio 1/1,4) [7]. En effet, cette prédominance féminine a été notée dans 66% des cas de notre étude. L'adénome pléomorphe se présente sous forme d'une tumeur arrondie ou fusiforme, indolore, sa croissance est lente et se fait par poussées [7]. A la palpation, la tumeur peut perdre son caractère régulier, sa consistance est variable: ferme, molle ou encore gélatiniforme, ce qui ne permet pas de préjuger de sa malignité [7]. Le traitement de l'adénome pléomorphe est chirurgical [7]. En effet tous les patients de notre travail avaient bénéficié d'une sous maxillectomie. Les kystes du cou sont des malformations d'origine embryologique peu fréquentes. On distingue les malformations médianes des malformations latérales, qui peuvent être ou non, d'origine branchiale [11]. Pour Piquet et Burny [11], les kystes représentaient 2% des tumeurs du cou, contre 4,76% des tuméfactions de la région sous mandibulaire dans notre série. Les tumeurs vasculaires survenant dans les glandes salivaires principales sont presque toujours hémangiomateuses. Les hémangiomes de la glande sous mandibulaire sont moins fréquents que ceux de la glande parotide [12]. McMenamin avait rapporté 13 cas d´hémangiomes de la glande sous mandibulaire avec revue de la littérature [12]. Son diagnostic chez l'adulte devient assez difficile, car il est rare et aucun examen non-invasif ne le confirme [12].

Dans notre série l'hémangiome avait été diagnostiqué chez un seul patient, le traitement avait consisté en une sous maxillectomie avec un examen extemporané pour un ganglion satellite qui avait révélé une inflammation sans signes de malignité. Le fibrolipome est peu fréquent au niveau de la région sous mandibulaire, c'est une tumeur bénigne, bien limitée, de siège sous cutané le plus souvent [13]. Selon Lancomme, les lipomes apparaissent sans préférence pour un sexe, et leur fréquence est maximale entre 40 et 60 ans [13]. Dans notre étude, il a été diagnostiqué chez un seul patient de 46 ans. Le traitement est l'exérèse par cervicotomie, ce geste est dicté par l'incertitude diagnostique et la crainte de laisser passer une affection maligne [13]. Les tumeurs de la glande sous mandibulaire ne représentent que 8 à 10% des tumeurs des glandes salivaires [7]. La part relative des tumeurs malignes est plus importante dans cette localisation qu'au niveau de la glande parotide.

Une analyse traitée sur une période de 10 ans a permis de montrer que 66% des tumeurs étaient bénignes et 34% étaient malignes [7]. Dans notre série nous avions noté 2 cas de tumeurs malignes, soit 29% de la pathologie tumorale, représentés par un cas de carcinome épidermoide et un cas de lymphome. Le traitement des tumeurs malignes des glandes salivaires doit faire discuter la chirurgie, la radiothérapie et la chimiothérapie [14]. Le carcinome épidermoide représente 7% des tumeurs malignes des glandes salivaires, c'est une tumeur rare du sujet âgé. La conduite à tenir pour notre cas avait consisté en une cervicotomie complétée par un curage ganglionnaire, avec radiothérapie et chimiothérapie. Établir une différence entre une tumeur primitive des glandes salivaires ou métastase intraglandulaire d'une tumeur extra-salivaire est essentielle pour le traitement et le pronostic [14]. Une telle distinction est souvent difficile pour certains types histologiques, notamment les carcinomes épidermoïdes. La localisation des métastases au niveau de la région sous mandibulaire est intraglandulaire dans 40% des cas, et intraganglionnaire dans 60% des cas [7]. La plupart des métastases localisées au niveau des glandes salivaires ont pour origine un mélanome (40%) ou un carcinome épidermoïde (33%) de la région [14]. Pour le cas de notre série, l'examen histologique avait confirmé la métastase par un carcinome épidermoïde. La plupart des lymphomes des glandes salivaires sont des lymphomes non hodgkiniens (85%) [15]. Près de 40% des lymphomes malins de la tête et du cou touchent les glandes salivaires [15]. Dans notre étude, un lymphome T avait été diagnostiqué chez un patient de 68 ans.

## Conclusion

Les tuméfactions sous mandibulaires sont relativement rares et regroupent des entités histologiques très variées. Le bilan radiologique est primordial, il permet souvent d’évoquer le diagnostic, dont la confirmation est indispensable par un examen histologique extemporané ou de la pièce d'exérèse. Le protocole thérapeutique dépend de la nature bénigne ou maligne de la tuméfaction. La technique chirurgicale doit être adaptée à chaque type histologique. L’évolution des traitements actuels et le développement des techniques mini-invasives assistées par radiographie telles que la lithotritie ou la sialendoscopie sont d'utilisation croissante dans les pays développés avec de rares effets secondaires.
